# Challenges in clinical and laboratory diagnosis of androgen insensitivity syndrome: a case report

**DOI:** 10.1186/1752-1947-5-446

**Published:** 2011-09-08

**Authors:** Caroline OA Melo, Daniela M Silva, Aparecido D da Cruz

**Affiliations:** 1 Pontifícia Universidade Católica de Goiás, Departamento de Biologia, Núcleo de Pesquisas Replicon, Goiânia, Goiás, Brazil; 2 Pró-Reitoria de Pós-Graduação e Pesquisa, Mestrado em Genética, Pontifícia Universidade Católica de Goiás, Goiânia, Goiás, Brazil; 3 LaGene, Laboratório de Saúde Pública Dr Giovanni Cysneiros (Lacen), Laboratório de Citogenética Humana e Genética Molecular, Secretaria Estadual de Saúde de Goiás, Goiânia, Goiás, Brazil; 4 Departamento de Biologia Geral, Instituto de Ciências Biológicas, Universidade Federal de Goiás

## Abstract

**Introduction:**

Androgen is a generic term usually applied to describe a group of sex steroid hormones. Androgens are responsible for male sex differentiation during embryogenesis at the sixth or seventh week of gestation, triggering the development of the testes and penis in male fetuses, and are directed by the testicular determining factor: the gene *SRY *(sex determining region on Y chromosome) located on the short arm of chromosome Y. The differentiation of male external genitalia (penis, scrotum and penile urethra) occurs between the 9th and 13th weeks of pregnancy and requires adequate concentration of testosterone and the conversion of this to another more potent androgen, dihydrotestosterone, through the action of 5α-reductase in target tissues.

**Case presentation:**

This report describes the case of a teenage girl presenting with a male karyotype, and aims to determine the extension of the mutation that affected the AR gene. A Caucasian girl aged 15 was referred to our laboratory for genetic testing due to primary amenorrhea. Physical examination, karyotype testing and molecular analysis of the androgen receptor were critical in making the correct diagnosis of complete androgen insensitivity syndrome.

**Conclusions:**

Sex determination and differentiation depend on a cascade of events that begins with the establishment of chromosomal sex at fertilization and ends with sexual maturation at puberty, subsequently leading to fertility. Mutations affecting the *AR *gene may cause either complete or partial androgen insensitivity syndrome. The case reported here is consistent with complete androgen insensitivity syndrome, misdiagnosed at birth, and consequently our patient was raised both socially and educationally as a female. It is critical that health care providers understand the importance of properly diagnosing a newborn manifesting ambiguous genitalia. Furthermore, a child with a pseudohermaphrodite phenotype should always undergo adequate endocrine and genetic testing to reach a conclusive diagnosis before gender is assigned and surgical interventions are carried out. Our results show that extreme care must be taken in selecting the genetic tools that are utilized for the diagnosis for androgen insensitivity syndrome.

## Introduction

Androgen is a generic term usually applied to describe a group of sex steroid hormones. Androgens are produced primarily by a male's testes. However, some small amounts are also produced by the ovaries in females and by the adrenal gland in both sexes. Androgens are responsible for male sex differentiation during embryogenesis at the sixth or seventh week of gestation, triggering the development of the testes and penis in male fetuses, and are directed by the testicular determining factor: the gene *SRY *(sex determining region on Y chromosome) located on the short arm of chromosome Y. The differentiation of male external genitalia in the penis, scrotum and penile urethra occurs between the 9th and 13th weeks of pregnancy and requires adequate concentration of testosterone and conversion of this to another more potent androgen, dihydrotestosterone (DHT), through the action of 5α-reductase in target tissues [1]. The actions of testosterone and DHT require the presence of functional androgen receptors that, after signal from these hormones, activate the transcription of specific genes in target tissues. Thus, any abnormality in the production or action of androgens in a fetus 46, XY between the 9th and 13th weeks of pregnancy causes incomplete masculinization, resulting in male pseudo-hermaphroditism. In men, androgens also trigger the complex process of puberty, affecting the development of facial, body, and pubic hair, deepening of the voice, and muscle development. Physiologically, androgens regulate spermatogenesis and help maintain male reproductive functions [2].

**Figure 1:**
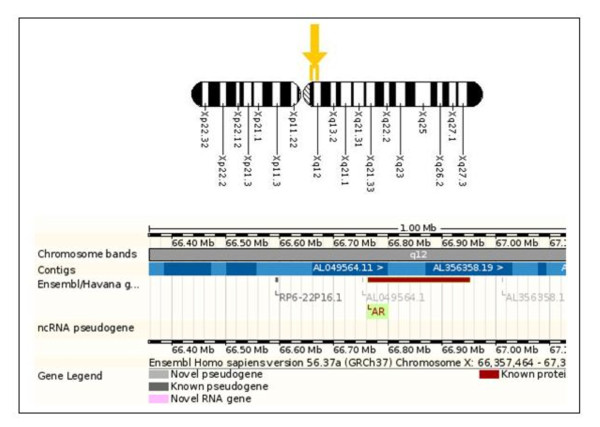
**Cytogenetic location of the *AR *gene, Xq11.2-q12, and molecular location on the X chromosome, base pairs 66,680,598 to 66,860,843**.

The *AR *gene is a protein-coding gene located at Xq11.2-q12. It spans over 90 kb and codes for a protein that functions as a steroid-hormone-activated transcription factor (Figure 1). The androgen receptor (AR), like other members of the nuclear receptor superfamily, has three major functional domains. The AR is characterized by a modular structure consisting of four functional domains: an N-terminal domain (NTD), a DNA-binding domain (DBD), a hinge region, and a ligand-binding domain (LBD; in this case, the ligand being an androgen) [3].

The complete form of androgen insensitivity syndrome (CAIS) is relatively rare. In childhood, the most common clinical presentation is the presence of bilateral inguinal hernias. Individuals not diagnosed during childhood are detected after puberty because of primary amenorrhea. Patients with the complete form of AIS have female external genitalia, an absence or thinning of pubic hair and the absence of a uterus [4].

The AR NTD is relatively long and displays the greatest sequence variability among nuclear receptors. It is very flexible and displays a high degree of intrinsic disorder. In addition, the NTD has a variable number of homopolymeric repeats, the most important of which is a polyglutamine repeat that ranges from eight to 31 repeats in normal individuals, with an average length of 20 base pairs [5].

The DBD is centrally located in the AR and it is the most conserved region within the nuclear receptor family. This region has nine cysteine residues, eight of which are involved in forming two zinc fingers, and a C-terminal extension. The first zinc finger, most proximal to the NTD, determines the specificity of DNA recognition, whereas residues in the second zinc finger are involved in AR dimerization. Two AR monomers in a head-to-head conformation bind as a homodimer to androgen response elements which are direct or indirect repeats of the core consensus 5'-TGTTCT-3' or more complex response elements with diverse arrangements of AREs. The C-terminal extension is important for the three-dimensional structure of the DBD and it plays a role in mediating the AR selectivity of DNA interaction [6].

The hinge region has long been considered to be a flexible linker between the DBD and LBD in the AR. More recently, however, this region was shown to be involved in DNA binding as well as AR dimerization. It was suggested that the hinge region also acts to attenuate transcriptional activity of the *AR *gene [7].

Mutations in the *AR *gene cause X-linked androgen insensitivity syndrome (AIS) characterized by androgen unresponsiveness, which affects proper male sexual development both at embryogenesis and at puberty. As a genetic disorder, AIS presents problems to affected people and their families, and is a major medical challenge for health providers. This impaired response to androgen results from an incapacity or reduced capacity of the AR to transactivate androgen-responsive genes in target cells, and leads to abnormal differentiation and development of male internal and external genitalia, leading to male pseudo-hermaphroditism [1].

Most mutations on the *AR *gene result in AIS (OMIN #300068). Depending on the extent of the AR defect, the phenotype can vary. Three forms are classified, complete (CAIS), partial (PAIS), and mild (MAIS). All forms of AIS are X-linked traits inherited as a recessive disorder. Despite a normal male karyotype (46, XY), individuals affected with CAIS (also known as testicular feminization syndrome) have female external genitalia, blind vaginas, an absent uterus and female adnexa, female breast development, and abdominal or inguinal testes. Partial androgen insensitivity results in hypospadias and micropenis with gynecomastia [8]. Patients usually come to medical attention during the neonatal period because of inguinal hernia and/or ambiguous genitalia, or at puberty because of primary amenorrhea associated with normal breast development and reduced pubic hair. In contrast, PAIS is a heterogeneous condition and covers a wide spectrum of undervirilization situations resulting in different degrees of ambiguous external genitalia, ranging from an almost complete form to phenotypic males with isolated azoospermia [9].

The aim of the investigation reported here was to provide a genetic diagnosis of a teenage girl with normal male karyotype using fluorescence *in situ *hybridization (FISH) and PCR in order to determine the nature and the extent of the mutation that affected the *AR *gene.

## Case presentation

A 15-year-old Caucasian girl was referred to a laboratory in Goiânia, Goiás, Brazil, for genetic testing due to primary amenorrhea. Her medical history included removal of an abdominal mass as a newborn. The tissue removed was referred to as an umbilical hernia. Her G-band karyotype revealed a diploid set of chromosomes, including 22 pairs of homologous autosomes and one pair of sex chromosomes, compatible with a 46, XY male chromosome complement.

The geneticists at our laboratory concluded that the mass withdrawal from the abdomen of our patient was, in fact, testes, and that our patient had a condition known as cryptorchidism; a reproductive change characterized by a failure of the movement of one or both testes from the abdominal cavity to the scrotum.

We performed PCR and FISH to verify mutations of the exons 1, 4, 6, 7 and 8 of the *AR *gene and to detect the *AR *gene, respectively. We prepared a culture of T lymphocytes in RPMI 1640 medium, supplemented with 20% fetal calf serum and 2% of phytohemagglutinin. Metaphasic preparations were made by conventional methodology. The slides for FISH were prepared with a micropipette, with about 15 μL of the material set. Only slides of good quality (in terms of metaphase) were selected by phase contrast microscope, and were subjected to FISH using the LSI Androgen Receptor SpectrumOrange (Xq12) probe (Vysis, Abbott Park, IL, USA). For PCR, primers were used for exons 1, 4, 6, 7 and 8 of AR [10].

**Figure 2:**
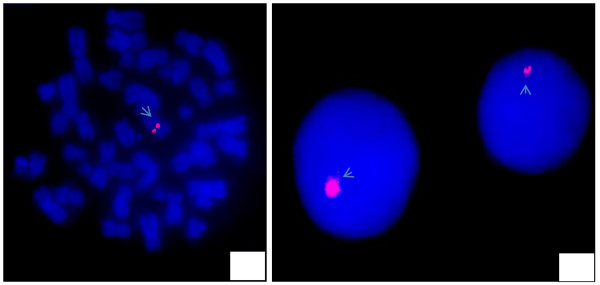
**Hybridization signals for the LSI androgen receptor probe, indicating the presence of the *AR *gene on the nuclei of a 15-year-old patient affected with androgen insensitivity syndrome (AIS)**.

*In situ *hybridization with the LSI AR probe indicated the presence of the gene in all analyzed cells. However, genomic DNA extracted from peripheral blood leukocytes assessed by PCR revealed coding sequence abnormalities for the *AR *gene, which lacked exons 1 to 7 indicating large deletion spanning the proximal region of the gene. Figure 2 shows hybridization signals in both interphase and metaphase nuclei.

## Conclusions

**Figure 3:**
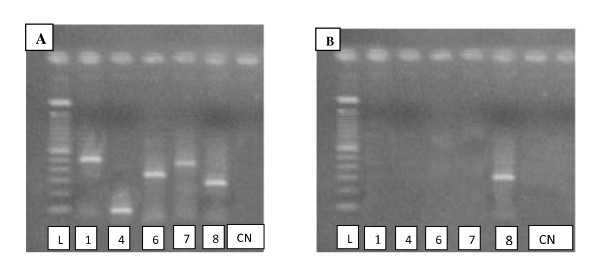
**Images of polymerase chain reaction products on a 2% agarose gel**. The first image (A) shows a gel from a normal patient with the five exons analyzed. The second image (B) shows a gel from our patient, who had only exon 7. CN = negative control; L = ladder of 100 bp; 1 = exon 1 with a size of 528 bp; 4 = exon 4 with a size of 172 bp; 6 = exon 6 with a size of 378 bp; 7 = exon 7 with a size of 516 bp; 8 = exon 8 with a size of 289 bp.

**Figure 4:**
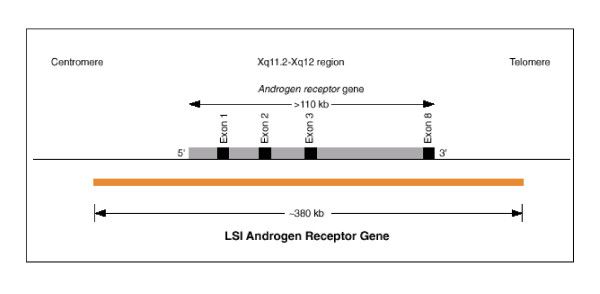
**Map of probe and exons amplified by polymerase chain reaction showing the problem with fluorescence *in situ *hybridization (FISH) and lack of exons 1 to 7 on the *AR *gene**.

The results reported in this paper underline the fact that great care must be taken when selecting genetic testing tools to be utilized to reach the proper diagnosis for AIS. If a child reaches his or her teenage years undiagnosed due to clinical challenges presented by ambiguous genitalia it further complicates matters, as there could be several genetic events subjacent to that outcome, ranging from total or partial deletion of the gene to point mutation, that efficiently silences the gene and therefore leads to AIS. Here, we report that FISH alone was not able to properly diagnose our patient, despite the proximal deletion within the AR observed on PCR (Figure 3). This result could be explained by the size of the probe used (380 kb), which was bigger than the AR gene (90 kb), indicating that the deletion of some exons within the gene was not large enough to prevent probe hybridization (Figure 4) [11]. Thus, in our patient's case, the PCR assay was able to confirm the diagnosis for our patient with a history of abdominal mass removal as a newborn who had a chromosomally normal male karyotype. However, due to complex chromosome aberrations or other genomic mutations, other molecular tools to detect and define DNA mutations, such as DNA sequencing, may be required to properly reach the conclusion of a diagnosis of AIS.

Sex determination and differentiation depend on a cascade of events that begins with the establishment of chromosomal sex at fertilization and ends with sexual maturation at puberty subsequently leading to fertility. Mutations affecting the *AR *gene may cause either complete or partial AIS. The case of our patient reported here is consistent with CAIS, misdiagnosed at birth, and she was consequently raised socially and educationally as a female. It is critical that health care providers understand the importance of properly diagnosing a newborn with ambiguous genitalia. Prompt evaluation of both clinical and genetic findings is crucial to determine proper gender assignment and the detection of life-threatening conditions [12]. Furthermore, a child with a pseudohermaphrodite phenotype should always undergo adequate endocrine and genetic testing for a definitive diagnosis before gender is assigned and surgical interventions are carried out. Inadequate investigation may result in inappropriate gender assignment in infancy with possible inferences on outcome [13].

The presentation of a patient, and specifically a neonate, with abnormal genital development represents a difficult diagnostic and therapeutic challenge. Referral to a center with experience in the diagnosis and management of disorders of sexual development is advised, where an emphasis should be placed on psychological and genetic counseling [14,15].

## Consent

Written informed consent was obtained from the patient's next-of-kin for publication of this case report and any accompanying images. A copy of the written consent is available for review by the Editor-in-Chief of this journal.

## Competing interests

The authors declare that they have no competing interests.

## Authors' contributions

COAM was involved in collating the information, reviewing the literature, and preparation of the manuscript. DMS was involved in collating information regarding the case and getting informed consent from our patient. ADC was involved in the review of literature and revising the manuscript critically. All authors read and approved the final manuscript.
